# Reconstruction of medium-size defects of the oral cavity: radial forearm free flap vs facial artery musculo-mucosal flap

**DOI:** 10.1186/s40463-021-00523-z

**Published:** 2021-12-03

**Authors:** Badr Ibrahim, Akram Rahal, Eric Bissada, Apostolos Christopoulos, Louis Guertin, Tareck Ayad

**Affiliations:** 1 Division of Otolaryngology-Head & neck Surgery, Université de Montréal, Hôpital Sacré Coeur de Montréal, Montreal, Canada; 2 Division of Otolaryngology-Head and Neck Surgery, Université de Montréal, Hôpital Maisonneuve-Rosemont, Montreal, Canada; 3 Division of Otolaryngology-Head and Neck Surgery, Université de Montréal, Centre Hospitalier de l’Université de Montréal, 1000 Saint-Denis St, Montreal, QC H2X 0C1 Canada

**Keywords:** Oral cavity, Locoregional flap, Free flap, RFFF, FAMM

## Abstract

**Background:**

The radial forearm free flap (RFFF) is the most commonly used flap for defects of the oral cavity. The facial artery musculomucosal (FAMM) is a safe and effective method to reconstruct medium sized defects of the oral cavity. No comparison exists between the FAMM flap and RFFF.

**Methods:**

1) Retrospective chart review from 2007 to 2016. 2) Cost difference analysis.

**Results:**

Thirteen FAMM flap cases and 18 RFFF met inclusion criteria. The FAMM flap showed a tendency to lower rates of return to the operating room (*p* = 0.065) as well as lower rates of complications not requiring return to the OR with 1 complication in 1 patient as opposed to 10 patients with 15 complications (*p* = 0.008). Also, FAMM flap had shorter operative times compared to the RFFF group (7.2HR and 8.9 HR respectively, *p* = 0.002). The average operative room related costs for a FAMM flap were 6510 CAD vs 10,703 CAD for RFFF (*p* < 0.0005). Speech and swallowing outcomes were similar (*p* > 0.05).

**Conclusion:**

The FAMM flap can be used for reconstruction of medium-size defects of the oral cavity with functional outcomes similar to the RFFF while decreasing the associated costs and morbidity.

**Graphical Abstract:**

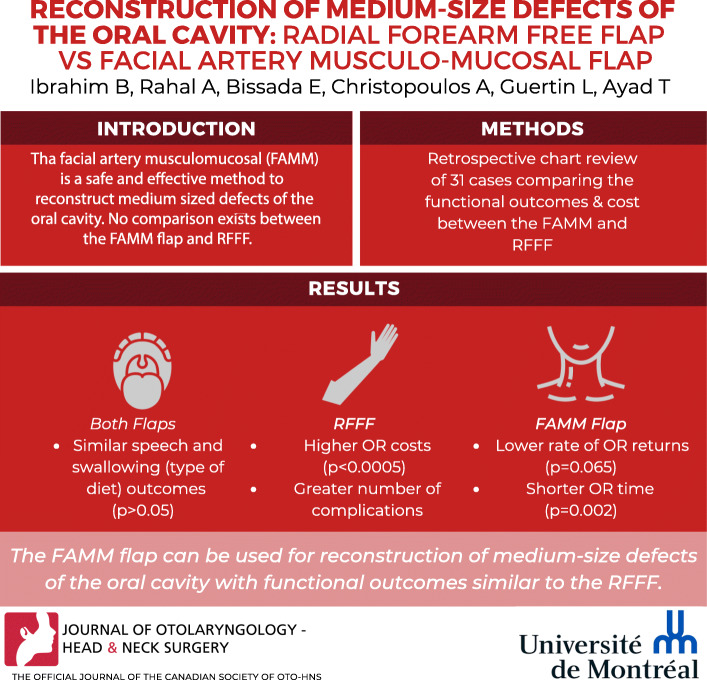

## Background

The modern reconstructive paradigm has evolved around the use of free tissue transfer preferentially for reconstruction of the oral cavity. The radial forearm free flap (RFFF) emerged as the workhorse of free flap soft tissue oral cavity reconstructions due to its versatility and capacity to reconstruct medium and large size defect in all subsites of the oral cavity [1]. The alternative to the RFFF is the anterolateral thigh (ALT) flap providing at times disproportionate bulk for the oral cavity. On the other hand, some locoregional pedicled flaps seem to achieve similarly appropriate reconstruction of soft tissue defects of the oral cavity. Good outcomes have been reported using the submental island flap, the supraclavicular flap and the facial artery musculo-mucosal (FAMM) flap [2–6].

In the last decade, an expansion of the indications and the development of the surgical techniques for these locoregional flaps has led to a slow but important paradigm shift in oral cavity reconstruction making way for these locoregional flaps as an alternative reconstructive method in selected cases. Indeed, accumulating evidence points towards a decrease in health care costs and resource utilization, as well as decreased operating times and reduced donor site morbidity with the use of some locoregional alternatives, with no compromise in surgical or functional outcomes [3–5, 7].

A particularly fitting option for the oral cavity is the FAMM flap. It has been successfully used for the reconstruction of complex oral cavity defects demonstrating an excellent safety profile [8]. Moreover, its ease of harvest, pliability, and its proximity to the site of defect make it a prime candidate for efficient trans-oral ablation and reconstruction of oral cavity defects. However, to this date, there is no data in the literature comparing the FAMM flap to the RFFF for oral cavity reconstructions. Therefore, we sought to compare the results of our tertiary academic referral center’s experience with the use of RFFF and FAMM flaps for medium-size defects of the oral cavity, comparing surgical and functional outcomes, as well as providing a cost difference analysis.

## Methods

### Data collection and analysis

**Fig. 1:**
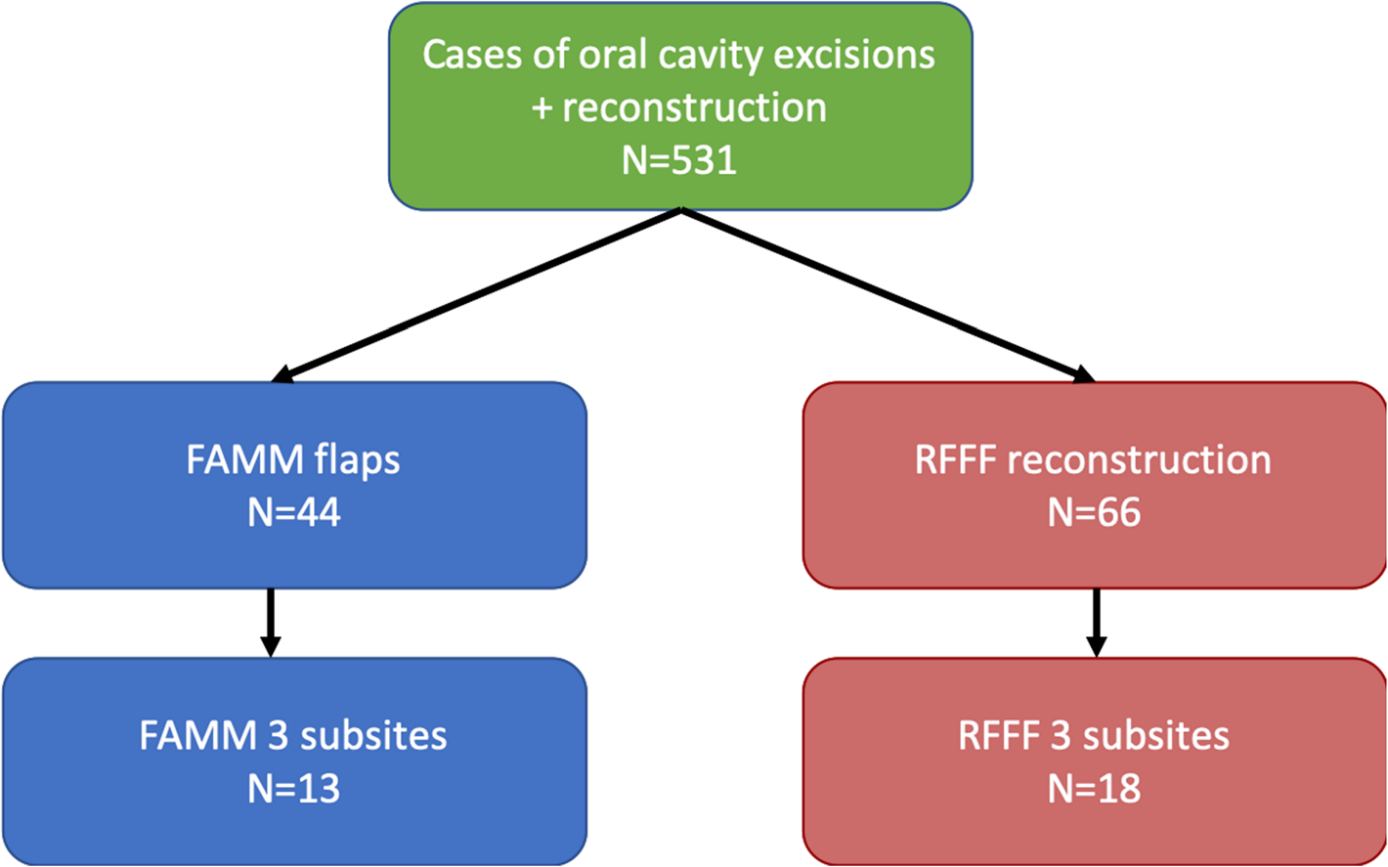
Case inclusion breakdown

Following institutional board review approval, we conducted a retrospective review of all patients who had undergone an oral cavity resection for which a FAMM flap or RFFF was selected for the reconstruction in one academic referral center from January 2007 to June 2016 inclusively. Operative notes were reviewed in detail to identify patients with oral cavity cancers who had a defect spanning at least three distinct subsites of the oral cavity or oropharynx for inclusion in the study (Fig. 1). We have chosen a cut off for defects spanning at least 3 subsites in an attempt to identify patients for which a RFFF would have likely been the reconstructive choice in many other centers, hence reducing the risk of bias related to size of the defect. Once included, patient’s medical charts were reviewed for demographic data as well as data pertaining to the perioperative hospital course following surgery, and subsequent outpatient visits. Pathology reports and imaging results were used to confirm staging and margins. Furthermore, details pertaining to speech, diet as well as dental rehabilitation were gathered from systematic evaluations by dedicated speech language pathologists, nutritionist and dentists.

All time intervals were calculated in days from the date of surgery. Follow up time was defined as the difference in days between the date of the surgery and the last recorded encounter with a member of the multidisciplinary team or the date of death as recorded in the hospital’s electronic chart system.

Functional status for speech and diet was assessed using the data from the latest recorded note by the speech language pathologist and nutritionist. Based on the information gathered in these notes the outcome for speech was classified into one of 5 standard categories defined in “Understandability of Speech” domain of the RTOG Performance Status Scale For Head And Neck Cancer Patient: PSS-HN questionnaire [9].

Data analysis was made by means of descriptive statistics. Comparative analysis between FAMM and RFFF were made using a two-tailed chi-squared test, with Fisher correction when indicated, for discrete variables and Mann-Whitney U test for continuous variables. Disease free survival was analyzed using the Kaplan Meier estimator and the equality of survival function was tested using the cox proportional hazard regression. The significance level was set at *p* < 0.05. All analysis were performed using StataSE 13.0.

### Cost difference analysis

The scheme for calculating the total costs of procedures is detailed in Table 1. Of note, the cost of surgery was calculated independently for the initial procedure plus every additional operative session (OR) required to address complications linked to the initial surgery. The sum of the costs of all ORs including the initial surgery make up the final value input in the formula of Total Cost. As well, if a secondary surgery required a re-hospitalization on the ward or in the ICU the cost of the hospitalization was added to the cost of the new initial hospitalization to input in the formula of Total Cost.

**Table 1:** Cost calculation scheme

Total cost = cost of each surgery + cost of hospitalization	
Cost of surgery	Cost of hospitalization
# of OR nurse * hourly rate* duration of surgery	# ward nights
Respiratory therapist hourly rate* duration of surgery	# Intensive care unit nights
Cost of anesthetist	
Cost of all surgeons involved per OR	
Cost of consumables	

All prices for consumables as well as the hourly rate of OR nurses and respiratory therapist were obtained from the supplies and accounting office of the operating room. The cost of surgeons and anesthetists were calculated using the ministry of health 2017 physician re-imbursements specific to Anesthesia, Plastic Surgery and Otolaryngology Head and Neck Surgery. Data pertaining to the cost of hospitalization was obtained from the accounting office of the hospital and reflect the specific ICU and ward night’s fixed prices determined by the ministry of health in 2017. All values are in 2017 Canadian Dollars (CAD). The continuous variables describing the mean total costs, costs of surgery and costs of hospitalization were compared between groups using the Man Whitney U test.

## Results

Demographic data and data pertaining to the size of the primary tumor and the surgical defects to be reconstructed are shown in Table 2 and Table 3. While 3 patients in the RFFF group had positive margins, 1 patient presented carcinoma in situ and two others had high grade dysplasia at the final pathology. In comparison, in the FAMM group, 1 patient had a positive margin and 3 had carcinoma in situ at the final pathology. The disease-free survival was similar in both groups. (*p* = 0.312). The available data from 7/13 FAMM flap patients and 15/18 RFFF patient regarding flaps surface area show a higher surface area for RFFF (27 cm^2^ VS 40 cm^2^; *p* = 0.005). The FAMM flaps covered defects created by the removal of specimens measuring from 3x3x2 cm to 6x4x2 cm in pathology after fixation. For the RFFF, the range spanned from 4 × 3,7 × 2 cm to o7,4 × 5,4 × 3,5 cm. All dimensions are detailed in Table 3. The FAMM flaps width ranged from 2 to 3 cm and a maximal length of 12.5 cm was harvested. A tracheostomy was performed for all patients in both groups. Subsites involved by the defects and oncological outcomes are detailed on a case by case basis in Table 3.

**Table 2:** Demographic data

Variable	FAMM (***n*** = 13)	RFFF (***n*** = 18)	***p*** value
Gender (Male)	8	10	0.739
Age	66	62	0.306
ASA class			0.464
1	0	1	
2	8	13	
3	5	4	
Active tobacco	4	7	0.641
1 ° Reconstruction	12	17	1
Follow up in months	24.5	37	0.901
T classification			0.625
is	1	1	
1	7	6	
2	3	8	
3	0	1	
4	2	2	
N classification			1
0	9	13	
1	1	1	
2a	0	0	
2b	2	2	
2c	1	2	
3	0	0	
Adjuvant Radio Therapy	4	9	0.462
Mandibulectomy^a^	7	7	0.98
Positive margin	1	3	0.365
Number of patients with 4 subsite defects	4	3	0.354

^a^ All were marginal mandibulectomy except 2/7 were segmental mandibulectomy in the FAMM group. The FAMM flap was used for soft tissue coverage in these 2 cases while the bone defect was reconstructed independently

**Table 3:** Oncological details by case

	Lip	Floor of mouth	Tongue	Alveolar crest	Cheek	Retromolar trigone	Anterior pilar	Tongue base	Soft palate	Dimensions of fixated pathology specimen in cm	Stage & TNM	Margin status	Adjuvant therapy	Follow up in months	Disease free survival in months
**Radial forearm free flap**															
Case 1		Y	Y		Y		Y	Y		7.4 × 5.4 × 3.5	4a (T3N2bM0)	Negative	No	4	2
Case 2		Y	Y	Y						5.5x4x1.5	4a (T2N2cM0)	Close	60 Gy + cisplatin	10	6
Case 3		Y	Y (V)	Y*		Y				5.5 × 4.5 × 2	2 (T2N0M0)	In situ	No	52	---
Case 4		y	Y	Y*						5 × 4.5 × 1.5	2 (T2N0M0)	Negative	No	51	---
Case 5		Y	Y (V)	Y						5.5 × 4.×1	1 (T1N0M0)	Negative	No	40	---
Case 6		Y	Y	Y		Y		Y	Y	6.5 × 6.5 × 3	1a (T1N0M0) **	Negative	No	12	8
Case 7		Y	Y	Y*						5x5x1.5	3 (T1N1M0)	Close	66 Gy	34	---
Case 8		Y	Y (V)	Y*						5x5x2	4a (T4aN2bM0)	Positive	No	6	0
Case 9				Y	Y	Y				5 × 4.4 × 1.7	1 (T1N0M0)	Negative	No	29	---
Case 10		Y	Y	Y*						6 × 4.7 × 1.3	2 (T2N0M0)	Negative	No	25	---
Case 11		Y	Y	Y						6.3 × 4.9 × 2.3	2 (T2N0M0)	Positive (negative on frozen)	66 Gy + cisplatin	18	---
Case 12		Y	Y	Y						4.5x4x1.5	1 (T1N0M0)	Negative	60 Gy	88	---
Case 13				Y	Y	Y				5.0 × 2.5 × 2.0	2 (T2N0M0)	Close	60 Gy	99	63
Case 14		Y	Y	Y						5.8 × 5.5 × 2	Is (TisN0M0)	Negative	NA	1	---
Case 15		Y	Y (V)	Y*						4 × 3.7 × 2	1 (T1N0M0)	Positive	66 Gy	67	0
Case 16		Y	Y	Y						8.5 × 4.1.5	2 (T2N0M0)	High grade dysplasia	66 Gy	39	---
Case 17		Y	Y	Y*						4 × 2.5 × 2	4a (T4aN2cM0)	Close	800 cGy	24	21
Case 18		Y	Y (V)		Y	Y		Y	Y	7.5x5x2	2 (T2N0M0)	High grade dysplasia	66 Gy	69	56
**Facial artery musculo mucosal flap**															
Case 1		Y	Y (V)	Y						5.5 × 3.8 × 2.5	4a (T4aN2bM0)	Positive	66 Gy	26	---
Case 2		Y	Y (V)	Y*			Y			5x3x1.5	4a (T4aN0M0)	in situ	No	35	26
Case 3		Y	Y (V)	Y*						3.5x3x1	1 (T1N0M0)	Negative	60	66	---
Case 4		Y	Y					Y		6x4x2	4a (T2N2bM0)	Close	66	70	6
Case 5	Y	Y		Y*						3x3x2	2 (T2N0M0)	Negative	No	10	3
Case 6		Y	Y (V)	Y*						4 × 1.5 × 2	3 (T1N1M0)	Negative	No	16	---
Case 7	Y	Y	Y (V)	Y						4 × 3.5 × 2	2 (T2N0M0)	in situ	No	17	6
Case 8		Y	Y (V)	Y*		Y				4.5x3x2.5	4a (T4aN2cM0)	Close	66	26	12
Case 9		Y	Y (V)	Y						MD	1 (T1N0M0)	Negative	No	23	---
Case 10		Y	Y (V)	Y						4.5 × 2.8 × 1	1 (T1N0M0)	Negative	No	18	---
Case 11		Y	Y (V)	Y*						4x3x1	2 (T2N0M0)	in situ	No	11	---
Case 12		Y	Y(V)	Y						5.7 × 3.4 × 1.4	2 (T2N0M0)	Negative	No	1	---
Case 13		Y	Y (V)	Y*						6x4x1.5	1 (T1N0M0)	Negative	No	2	---

Y subsite included in the defect

(V) ventral tongue involved

* Mandibulectomy required

** Radio-induced sarcoma

--- Patient was disease free at time of last follow up

*MD* missing data

As far as surgical complications are concerned, there was a statistically significant difference in the proportion of patients with postoperative complications not requiring return to the OR with only 1 patient in the FAMM group with 1 complication as opposed to 10 in the RFFF group with 15 complications (*p* = 0.008). The distribution of these complications is detailed in Table 4. Of note, donor site complications represented the most frequent complication in the RFFF group with 3 cases of tendinous exposure that healed by secondary intention, 3 cases of hypoesthesia of the thumb and 1 seroma. In comparison, immediate and delayed complications requiring a return to the OR were noted in 8 patients in the entire series, requiring 11 returns to the OR for complications related to the initial surgery. There was no statistically significant difference in the proportion of patients in each group requiring a return to the OR (*p* = 0.065). It is worth noting however, that in the 11 instances of return to the OR, 9 were from the RFFF group while only 2 were from the FAMM group. The details and distribution of these complications are showed in Table 5. Of note, the 2 patients in the FAMM flap group who needed to return to the OR where returning for planned second stage elective surgery. One patient needed pedicle sectioning for dental rehabilitation while the other needed pedicle sectioning for treating trismus preventing his full dental rehabilitation. None of the FAMM flap cases developed a cheek hematoma, abscess or parotitis. We did not need to abort a FAMM flap for any patient due to vessel injury/nodal disease in our series.

**Table 4:** Post-operative complications not requiring return to the OR

	FAMM (***n*** = 13)	RFFF (***n*** = 18)
Chyle leak	1	0
Salivary fistula	0	1
Flap dehiscence	0	3
Hematoma	0	1
Minor donor site complication*	0	7
Cervical wound dehiscence	0	1
ICU admissions	0	3

*Refers to complications managed conservatively (such as local wound infections, healing issues with the donor site related to the skin graft etc)

**Table 5:** Complications requiring return to the OR

	Number of return	Reason for return
**RFFF**		
Case 1	1	Elective cure of ankyloglossia
Case 2	1	Tracheostomy re-do 2 days post discharge for acute airway obstruction
Case 3	1	Lingual artery blow out
Case 4	1	Venous congestion of flap – cervical hematoma drained
Case 5	2	Venous congestion of flap– anastomosis re-do Venous congestion of flap – anastomosis re-do
Case 6	3	Arterial compromise – anastomosis re-do Cervical hematoma Venous congestion – cervical hematoma drained
**FAMM**		
Case 1	1	Elective pedicle sectioning for dental rehabilitation
Case 2	1	Elective pedicle sectioning for trismus preventing dental rehabilitation

With regards to closure at the donor site, primary closure at the donor site was achieved in 33% of RFFF cases while 92% of FAMM flap donor sites were closed primarily (*p* = 0.002). For the remainder of the RFFF cases, a split-thickness skin graft was required to complete the closure while a secondary closure of a segment of the donor site incision was favored for FAMM cases.

Regarding the dental status for the FAMM flap cases, 9 out of 13 patients were edentulous at baseline. From the remainder four, 3 required a molar tooth extraction for flap inset.

The average surgical time for FAMM cases was 7.2 h compared to 8.9 h for RFFF (*p* = 0.002) and anesthesia time was 8.5 h for FAMM and 10.7 h for RFFF (*p* = 0.000).

**Fig. 2:**
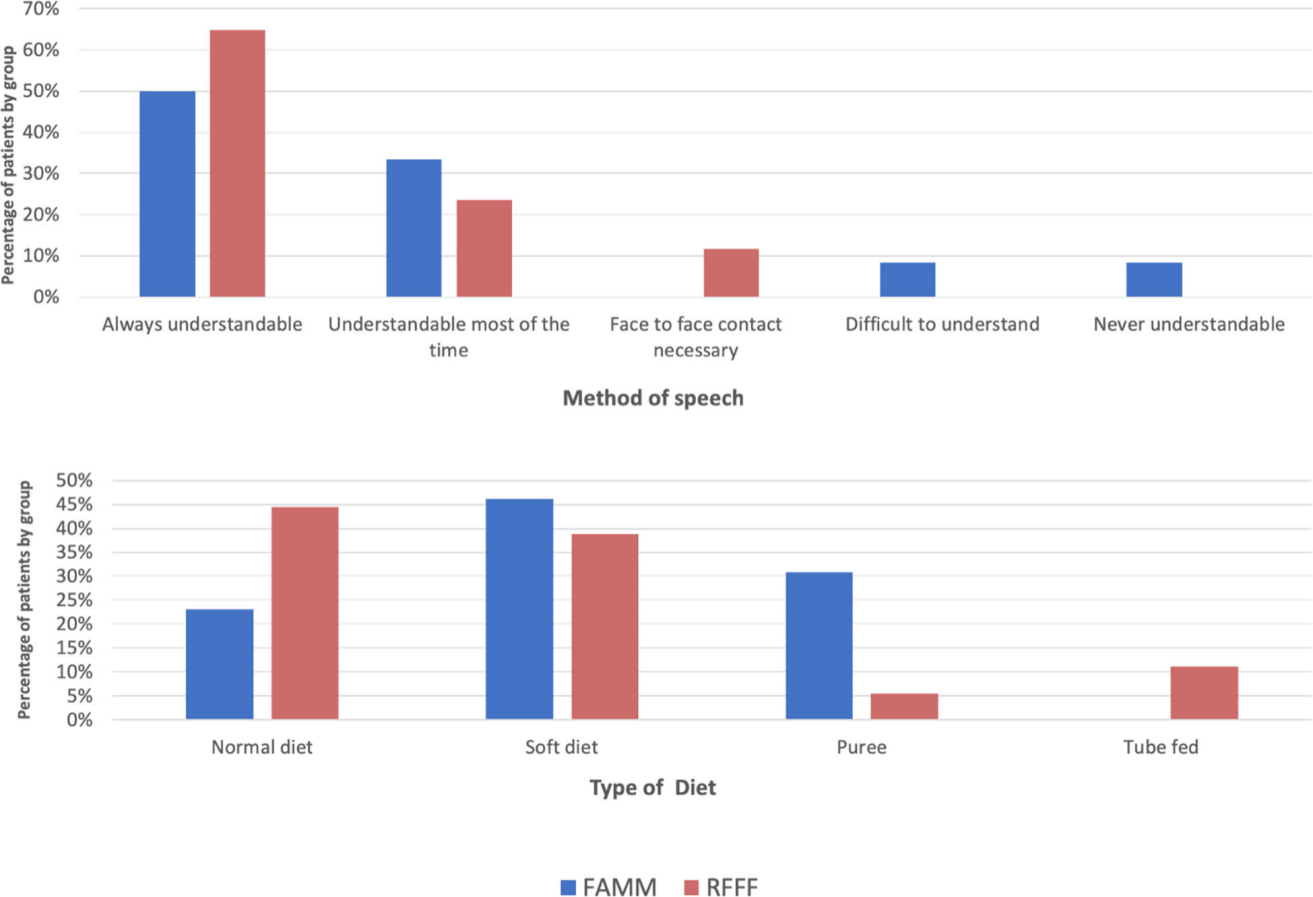
Speech (top) and feeding (bottom) outcomes distribution

No difference was noted between the groups for length of hospitalization (*p* = 0.717), time to decannulation (*p* = 1.00), duration of tube feeding (*p* = 0.449), speech (*p* = 0.314) or long-term feeding (*p* = 0.139) outcomes. The detailed distribution of speech and feeding outcomes is detailed in Fig. 2.

As far as the cost difference analysis is concerned, we have calculated an overall cost difference in the total average cost between the two reconstructive options at 11,059 CAD (*p* = 109). When broken down into its component, the average difference in the price of hospitalization was 6866 CAD (*p* = 0.482) while that of the cost of surgeries was 4193 CAD (*p* = 0.000). The details of the cost difference analysis are shown in Table 6.

**Table 6:** Cost difference analysis

Mean costs in CAD (median)	FAMM (***n*** = 13)	RFFF (***n*** = 18)	***p*** value
Cost of surgery	6510 (6477)	10,703 (9985)	0.000
Cost of hospitalization	17,679 (18,435)	24,545 (16,592)	0.482
Total cost	24,188 (25,292)	35,247 (26,594)	0.109

*CAD* Canadian Dollars

## Discussion

The rising cost of health care in the United states and Canada has become an undeniable reality that health care providers have to face and plan for in the delivery of high-quality health care. In the United States alone, a projected average increase in health care spending of 5.5% annually from 2017 to 2026 is predicted to outgrow GDP growth by 1% [10]**,** while a similar outgrowth of GDP has been observed in historic Canadian data from 1975 to 2017 [11]. In order to address this reality without compromising on the quality and standard of care we are committed to offer to our patients, it is useful to re-examine the indications and potential benefits, complications and costs of less invasive surgical alternatives.

Beyond the obvious benefits of eliminating the morbidity associated with the transposition of hair bearing skin in the oral cavity, the present study reveals that the use of the FAMM flap for medium-size defects of oral cavity was associated with approximately 20% decrease in surgical and anesthesia time.

We have demonstrated a clinically significant difference in number of returns to the OR with the use of FAMM flaps. Moreover, while all returns to the OR in the FAMM group were planned elective procedures, most of the RFFF patients returned to the OR on an emergent basis for serious acute complications. In fact, the returns to the OR in the FAMM flap groups were not true acute complications but rather second stage procedure to 1) section the pedicle to allow for oral rehabilitation and 2) to revise a check scar for trismus. We have chosen to discuss these as part of the “complications” as they are not necessary in every case, and they must be accounted for in the cost involved in doing a FAMM flap. There was no flap failure in any group but 2 patients in the RFFF group required revision of the anastomosis, which was successful. While no flap was lost in our series, these cases highlight the fact that free flap reconstruction, although associated with a generally excellent rate at success in excess of 90%, is still associated with a 3–25% rate of re-exploration as a consequence of micro-anastomosis [12–18]. We have noted a higher rate of hematoma requiring reintervention in the free flap group. While this can be a complication of any neck surgery, the more extensive and invasive work needed for free flap microanastomosis and transfer to the recipient site could explain the tendency observed in our series.

In well selected patients, we have demonstrated that functional results are similar between RFFF and FAMM flaps. The present study demonstrates comparable groups on the basis of the demographic and tumor specific data (Table 2) as well as the distribution of subsets involved in the reconstruction (Table 3). The latter has an important bearing on the potential for speech and swallow rehabilitation. The vast majority of the cases in both groups involved the floor of mouth, the tongue and the alveolar crest while a minority in each group overlapped with the oropharynx or the lip. Therefore, the functional outcomes were analyzed on a reasonably homogenous group of defects decreasing the risk of bias related to the subset involved. This functional outcome analysis is however limited by our small sample size. This precluded a sound multivariate analysis to understand the effect of the choice of reconstructive method on the outcome in isolation of competing factors. Such an analysis would require a much larger sample size given the number of variables that would need to be included in it. While this study is one of the first to compare FAMM and RFFF, future studies on this topic are needed with larger sample sizes, possibly multicentric studies, to evaluate in more details the impact on functional outcomes of each reconstructive method. Moreover, the retrospective nature of the study induces some potential bias in the data collection notably with regards to functional outcomes. Indeed, our SLP notes systematically indicated the functional outcome of speech at each visit, but the terms used did no match exactly those of the RTOG scale used to categorize the data. Therefore, a degree of bias can be induced by the interpretation of the data during data collection. In the case of the present study, all data was collected by a single author to minimize variable interpretation of qualitative data. However, future multicentric studies should aim to collect data prospectively.

**Fig. 3:**
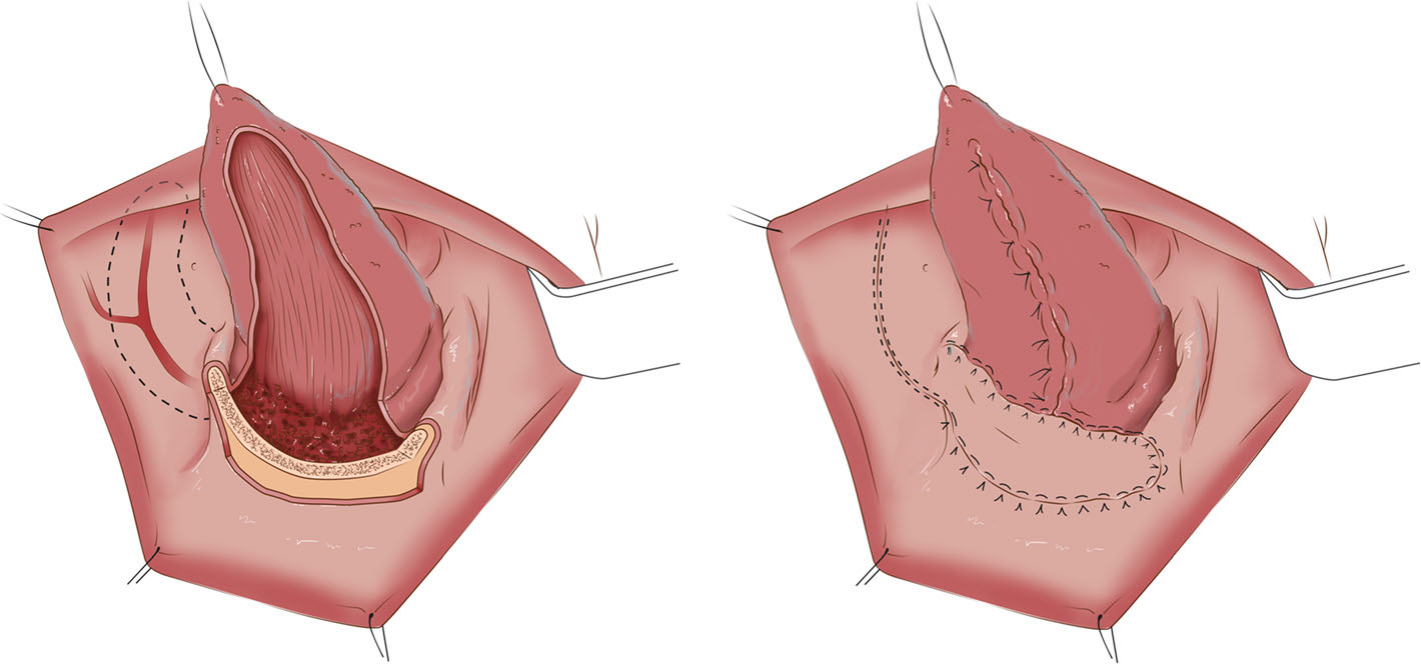
Depiction of a typical moderate size defect of the oral cavity suitable for a FAMM flap reconstruction. Left panel: the defect involves 3 adjacent subsites including up to one third of the mobile tongue. Right panel: The tongue defect can be closed primarily or left to granulate while the FAMM flap covers the alveolar crest and floor of mouth defect. The donor site is closed primarily

**Fig. 4:**
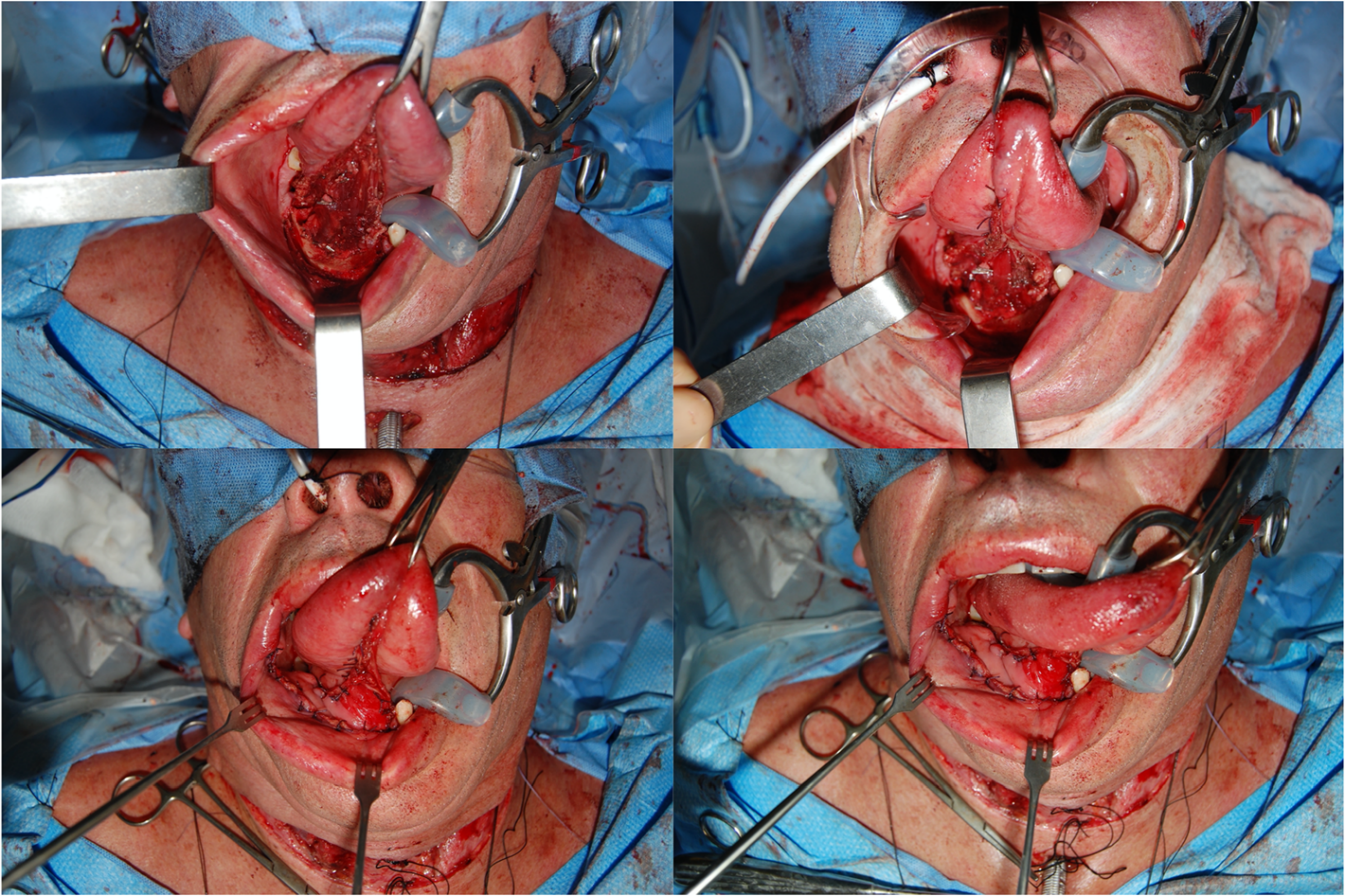
An example of multi-subsite medium size defect reconstructed by FAMM flap. Upper panel: Defect and primary closure of the ventral tongue to use th FAMM flap in the reconstruction of the remaining subsites. Lower Panel: Defect following FAMM flap reconstruction

Patients in the RFFF group had an average flap surface area that was larger than those in the FAMM flap group. However, a difference in flap size does not imply a difference in defect size in our group of patients. In the FAMM flap group, we tended to close the tongue primarily or by secondary intention while the FAMM flap was used to reconstruct the remaining subsites involved (Fig. 3). In the RFFF group, we used the extra length provided by this flap to reconstruct tongue defects in addition to the other subsites. Hence, our ideal medium size defects of the oral cavity suitable for FAMM flap reconstruction should not involve more than a third of the mobile tongue to allow primary closure of this subsite or healing by secondary intention. Moreover, if the lesion and its associated defect extended significantly to the contralateral side, we would favor a free flap to ensure the extra tissue provided by the free flap can reach far into the contralateral side. An example is shown in Fig. 4.

Due to the retrospective nature of our study, we could not measure specifically the defect sites. But it is safe to infer that defects were similar in size based on the similar T classification distribution between the groups. As well, by excluding all FAMM and RFFF cases that did not meet the criteria of a “3 subsites defect”, we have created an extra condition to ensure the indications for a FAMM or RFFF would be overlapping as much as possible.

From a health economics perspective, the cost difference analysis presented in this study shows a significant OR cost saving associated to the use of the FAMM flap. Free flap surgery is performed by a dual surgical team approach at our institution. While this approach has the merit of providing a setting for consultants to share ideas and expertise on a same case, it results in an obvious increase in the cost of the initial surgery. Moreover, the longer operative times and the presence of more nursing and paramedical staff for setting up the case and assisting two teams all contributed to increasing the price of the initial surgery. Of note, in the cases of FAMM flap, there was no specific code that could be billed for the reconstructive part of the surgery. Indeed, in the absence of a free flap performed by a second surgeon specifically, the price of the ablative and reconstructive portions of the surgery are bundled and determined based on the duration of the surgery. Hence, the absence of a specific code for the FAMM flap can further contribute to creating a price gap for the cost of surgery. This price gap is further compounded by the higher tendency to return to the OR in the RFFF group. Furthermore, while the number of nights of hospitalization did not vary significantly between the two groups, the average price of hospitalization was markedly elevated in the RFFF group. This is due to a subgroup of patients in the RFFF group requiring transfer to the intensive care unit (ICU) while no patient in the FAMM flap group was transferred to the ICU.

Our cost difference analysis was carefully designed to include as many direct and indirect costs related to the choice of the reconstructive method, a few caveats must be pointed out. Our institution has developed a post-operative monitoring protocol which allows patients with free flap reconstructions to be sent directly to a regular post-operative head and neck oncology ward. This has a major impact on the cost of hospitalization as it decreases the numbers of nights spent in the ICU hence making the cost difference less important at our institution compared to institutions where free flaps are routinely sent to the ICU at least for the first 24 h. Also, when calculating the price of the hospitalization for the RFFF group, the added nursing time dedicated to the monitoring of the flap following our protocol could not be accounted for precisely due to a lack of quantification by our institution’s accounting office of the extra personnel requested *specifically* for the flap monitoring as opposed to other activities on the ward. This results in an underestimation of the cost of hospitalization of the RFFF group. Moreover, an in-depth analysis of preoperative work up costs, and costs of postoperative follow up visits, cost of care for recurrences, and cost of follow up with allied health professionals for each type of reconstructive method was not provided. Also, due to some difference in local practice habits, the use of certain OR personnel (for e.g. respiratory therapist) or the number of personnel involved in each case may vary and limits the applicability of this cost difference analysis outside of the setting in which it was conducted. For instance, in this study an average hourly rate of 31.63 CAD was considered for the presence of respiratory therapist. This represents on average an added 281.5 CAD per RFFF case and 227.7 CAD per FAMM case. However, the 53.8 CAD difference in the average cost difference between RFFF and FAMM due to the respiratory therapist’s salary does not invalidate our findings even in a different setting where respiratory therapists are not routinely involved in the OR. Indeed, the main driver of cost difference was not in the cost of surgery itself rather than in the cost of hospitalization.

The FAMM flap is a useful versatile and safe option for the reconstruction of oral cavity defects but it has some technical limitations that should be considered when choosing a reconstructive option. Notably, in fully dentate patients it may require the extraction of a molar tooth for inset. While most of our patients in this series were edentulous at baseline, the dental status of the patient should be taken into account when considering a FAMM flap. The decision to extract a tooth should be weighed against the morbidity of the RFFF and discussed in conjunction with the patient in cases were both flap are acceptable options. Moreover, it may require pedicle sectioning for proper dental rehabilitation in up to one third of cases [8]. However, some modifications have been described that allow inset with no obstructing pedicle hence obviating the need for a potential second surgery while allowing for dental rehabilitation [19, 20].

A review of the literature demonstrates that locoregional flaps in head and neck reconstruction are undergoing a revival with multiple studies in the 5 years demonstrating the safety, functional, oncological and financial efficiency of locoregional flaps when compared to free flaps [3–5, 7, 12, 21]. Yet none of these studies addressed the comparison between the RFFF and the FAMM flap. However, the RFFF has been evaluated against the supraclavicular flap [3, 5] and the submental island flap [4, 21]. These studies have demonstrated a tendency to decreased morbidity at the donor site with similar functional outcomes as with the RFFF [4], as well as decreased operating times and hospital costs [6]. As well Paydarfar et al. have demonstrated that the use of the submental island flap in the oral cavity specifically was associated to shorter hospital stays, decreased recipient and donor site morbidity and similar functional outcomes as the RFFF [5]. Also, Forner et al. [21] have published similar results as ours when comparing the submental island flap to the RFFF in terms of operative time, hospital stay and cost analysis using a similar method of cost difference analysis. Contrasting our results however, they found a cost difference with regards to the hospitalization specifically. The patients undergoing RFFF in that study spent a minimum of 1 night in the ICU as part of the routine post-operative care. This could explain a wider gap between their RFFF and locoregional flap group compared to the present study. More generally, a tendency to cost reduction with the use of locoregional flaps in oral cavity and oropharyngeal reconstructions had been reported with different locoregional flaps [7].

## Conclusion

The use of the FAMM flap for reconstructing medium-size defects in selected patients is associated with lower costs, shorter OR time, similar functional outcomes and a tendency to lower complication rates. A nuance should therefore be introduced in the framework of thought of the reconstructive surgeon to include, in selected medium size defects, this locoregional flap as an alternative to the RFFF.

## Data Availability

The datasets generated and/or analyzed during the current study are not publicly available due restriction by our institutional ethics review board but can be made available from the corresponding author on reasonable request with approval of the ethics board for data sharing.

## Abbreviations

RFFF: Radial forearm free flap
FAMM: Facial artery musculomucosal flap
ALT: Antero lateral thigh flap
OR: Operating room
ICU: Intensive Care Unit
RTOG: Radiation Therapy Oncology Group
CAD: Canadian Dollar
GDP: Gross Domestic Product
